# The Impact of the COVID-19 Pandemic on Food Distribution at Emergency Food Assistance Organizations in the Southwestern United States: A Qualitative Investigation

**DOI:** 10.3390/nu13124267

**Published:** 2021-11-26

**Authors:** Alexandra N. Castro, Mamie A. White, Ariun Ishdorj, Debbe Thompson, Jayna M. Dave

**Affiliations:** 1 USDA/ARS Children’s Nutrition Research Center, Department of Pediatrics, Baylor College of Medicine, 1100 Bates Street, Houston, TX 77030, USA; Alexandra.Castro@bcm.edu (A.N.C.); mawhite@bcm.edu (M.A.W.); dit@bcm.edu (D.T.); 2 Department of Agricultural Economics, Texas A&M University, 2124 TAMU, College Station, TX 77843, USA; aishdorj@tamu.edu

**Keywords:** food insecurity, food distribution, emergency food assistance organizations (EFAOs), food pantry, COVID-19

## Abstract

This study aimed to identify changes in food distribution operations at emergency food assistance organizations (EFAOs) during the COVID-19 pandemic. EFAOs across the Houston metro area, TX (human service centers and food pantries) as well as the Houston Food Bank (HFB) participated in the qualitative study. Data were collected via individual semi-structured interviews and focus group (December 2020–February 2021), and coded using semi-structured thematic analysis. Categories were pre-identified based on the interview questions. Direct quotes supported subcategories. Directors from 18 EFAOs were interviewed; 8 HFB leadership staff participated in a focus group. Four major categories of change due to COVID-19 included new safety measures, changes in food distribution process, changes in volunteerism and staffing, and changes in amounts of food distributed. This study helps identify susceptibilities in EFAOs’ food distribution chain should be addressed to manage future emergency food insecurity crises more effectively. An understanding of the changes/challenges incurred by EFAOs during the COVID-19 pandemic can inform policymakers to ensure local food distribution organizations are prepared to fill the needs during future a crisis of food insecurity.

## 1. Introduction

The COVID-19 pandemic has resulted in unprecedented increase in food insecurity fueled by an economic recession and increasing unemployment rates [1]. Estimates for 2021 place more than 42 million people at risk of food insecurity [2]. Many households experiencing food insecurity are not eligible for federal nutrition assistance programs and rely heavily on Emergency Food Assistance Organizations (EFAOs) to supplement their nutritional needs [2].

EFAOs are non-profit organizations that collect and distribute food to people in need. These include multi-functional human service centers (HSC) (those providing services in addition to food distribution such as temporary shelters, income or rent assistance, and other social assistance) and stand-alone food pantries (FP). EFAOs distribute foods acquired through donations from retail partners, individuals, and the Feeding America food bank network, of which the Houston Food Bank (HFB) is the largest in the nation [3]. Moreover, the Emergency Food Assistance Program (TEFAP) is the main program subsidizing nutritious food at no cost to EFAOs [4]. Feeding America and its partner food banks have documented a nationwide increase in food insecurity and food demand since the COVID-19 pandemic began in March 2020 [2]; the HFB reported an increase of more than twice the usual 88 million pounds of food distributed [5].

Operational considerations from the Centers for Disease Control and Prevention (CDC) encouraged EFAOs to take special precautions to help prevent the spread of COVID-19 among staff, volunteers, and clients and help ensure safe access to food for their clients [6]. With significant increases in the number of clients and food demand, decreases in the number and age ranges of volunteers, and having to adhere to the CDC’s operational considerations to mitigate the spread of COVID-19, food distribution has been challenging for the EFAOs. There exists a gap in the current literature about the ways EFAOs have adapted to the changing circumstances of the COVID-19 pandemic.

This research was part of a larger qualitative study with EFAOs in a large urban area in Texas. We present the qualitative findings reporting changes implemented in food distribution operations at EFAOs during the COVID-19 pandemic.

## 2. Methods

### 2.1. Design

This is an exploratory qualitative study conducted with the EFAOs in the Houston Metropolitan Area, TX, USA. The Institutional Review Board at Baylor College of Medicine approved this study.

### 2.2. Recruitment

Using convenience sampling, 18 EFAOs, including HSCs and FPs were recruited for the study from different regions of the Houston metro area to help ensure demographic diversity. Prospective participants were identified through the HFB network and Hunger Free Texans—a network of organizations surveying underserved communities in TX. Recruitment occurred via phone calls and emails. From each EFAO, the food pantry director (or equivalent role) was recruited, and written consent was obtained. Additionally, leadership personnel from HFB managing EFAO relationships were recruited for this study.

### 2.3. Design

Data were collected by two trained and experienced research coordinators between December 2020 and February 2021 via individual semi-structured interviews with EFAO staff and a focus group with HFB leadership. Interviews were conducted in English and digitally recorded using either Zoom [7], telephone, or written correspondence based on participant preference. One focus group discussion with HFB staff was also conducted and digitally recorded over Zoom to elicit background context regarding the operations of partnering EFAOs.

Interviews and the focus group discussion were guided by a script developed by the research team. For this study, the interview script focused on three main topics with open-ended non-leading questions exploring the impact of the pandemic—closures, changes in operations, and changes in food distribution (Table S1). Prompts and probes were used to elicit more details and to clarify and understand responses. Participants were also invited to share any additional comments not previously covered but thought to be relevant to the discussion.

### 2.4. Data Analysis

Data from the interviews were transcribed verbatim and analyzed using a hybrid approach with semi-structured thematic analysis, which includes both deductive and inductive methods to code transcripts [8]. Initially, a codebook was developed with pre-identified categories (a priori codes) based on questions from the interview script. Subsequently, emergent codes and sub-codes were added to the codebook after in-depth reading of the transcripts. Codes were routinely reviewed and refined, applying codes to one transcript at a time, and revising the codebook as new concepts emerged. Changes were discussed until a final consensus was reached. The codebook functioned as an audit trail and included key decisions regarding the addition, removal or merging of codes into categories. Focus group data were analyzed separately using the same methodology. To protect confidentiality, ID numbers beginning with HSC or FP were randomly assigned to each interview.

## 3. Results

### 3.1. Participant Characteristics

A total of 19 staff members from 18 EFAOs participated in individual interviews. At that point theoretical saturation was reached [9]. In total, 10 EFAOs were HSCs and 8 were stand-alone FPs. The majority of EFAOs (*n* = 14) were associated with private ministries; these included 7 HSCs and 7 FPs. Seventeen EFAOs (except one HSC) were associated with HFB. Of all EFAO interviews, 11 were conducted over Zoom, 5 by phone, and 3 via written correspondence. Additionally, 8 individuals participated in the HFB focus group discussion. EFAO interviews averaged 47 min; the focus group discussion lasted 55 min.

### 3.2. Interview Findings

Four major categories of change directly impacted by the COVID-19 pandemic emerged from the interviews: new safety measures, changes in food distribution process, changes in volunteerism and staffing, and changes in the amounts of food distributed (Table 1). Categories 1–3 had multiple distinct topics and were divided into subcategories.

#### 3.2.1. Category 1: Safety Measures Adopted

Major safety measures adopted by the EFAOs included social distancing, use of PPE (including masks), health screenings, and increased cleaning (Table 2). Less common safety measures, labeled “other” in Table 2, included addition of physical barriers, increased airflow, and quarantining items. Although virtual work for administrative staff was mentioned, no EFAO food distribution component arms reported being able to work remotely due to the nature of food distribution. Therefore, remote work was not included as a safety measure.

##### Social Distancing

All participating EFAOs reported adhering to social distancing guidelines—i.e., a minimum of 6 feet between people [7]. The most common social distancing practice was a drive-through food distribution model: *Now we require people to have their trunks or their tailgates open so there’s no face-to-face contact* (HSC7A). HFB also supported and encouraged this model.

However, not all facilities had the luxury of spare square footage. Alternate ways in which social distancing was promoted were having more shifts with fewer volunteers (…*from 2 shifts of volunteers on Thursday and Saturday distribution to 7 shifts of volunteers*—FP11M) or limiting the number of people in the building and expanded waiting areas to outdoor spaces (We limited the number of volunteers … so that we can space out—HSC1A).

Limited space (…we just don’t have a large parking lot and our space is limited…we would have a lot of traffic jams and it wasn’t very organized—HSC6A) and fewer volunteers (Doing the drive-through outside just took too many people—HSC2A) were challenges reported for maintaining drive-through distribution. 

##### Personal Protective Equipment (PPE)

All EFAOs reported using masks from the beginning of the pandemic. Five sites also employed glove use. 

Two HSCs expressed some difficulty acquiring PPE: Our regular vendor that we work with, we have to call them directly to be able to get the quantity that we need because they max out. We can only buy two boxes every month (HSC2A).

##### Health Screenings

For the purposes of this study, health screenings were defined as any questionnaire (verbal/written) and/or temperature check imposed with the purpose of identifying a risk of exposure to COVID-19 and/or associated symptoms.

Elven EFAOs implemented health screenings for staff and volunteers. The most common type of health screening was temperature checks (*n* = 9; 5 HSCs and 4 FPs): *All staff and volunteers are required to wear a mask, take their temperature and record in a temperature log when entering the building or working a drive thru distribution* (FP16M). Four EFAOs (2 HSC and 2 FPs) reported requiring health surveys. Of the 11 total sites requiring health screenings, two FPs required both a health survey and a temperature check.

##### Increased Cleaning

Of the 18 EFAOs, nine (seven HSCs and two FPs) reported increased frequency in surface cleaning practices. One stated: *We were disinfecting things. We use a steam cleaner (HSC5A). Additionally, two HSCs employed weekly professional sanitation services: I never had a cleaning service until COVID happened. My people can’t keep it that clean. We were able to before we got this busy but now I have to have a cleaning service that comes once a week and sterilizes everything on a Friday after everyone is gone* (HSC2A). They also reported being unable to purchase basic cleaning products and hand sanitizer. For example, one stated: *I bought all the wipes I could find and did not worry about sanitizer. We had a bunch of alcohol and we could make our own because at that point the sanitizer was at $50 per gallon* (HSC5A).

Moreover, five EFAOs (four HSCs and one FP) mentioned increases in access to hand sanitizer and frequency in handwashing: For our clients, we have a sanitizing station set up at the door so that they can use it before entering and shopping. We have a grocery-store layout, so we want to make sure that whatever they touch they are doing it with clean hands (HSC6A).

##### Less Common Safety Measures

Only one HSC and one FP placed physical barriers in their buildings to segregate staff/volunteers from clients*: I wrote a grant to help me buy lumber and plexiglass and I had those built, so we now have six desks that have plexiglass* (HSC2A). Two HSCs reported switching from walk-ins to appointment-only distribution. Two HSCs found alternate ways to increase airflow such as opening windows and doors since moving food distribution outdoors was not possible.

One HSC quarantined their foods to ensure food would be safe to handle: *Food has to sit for three days before it gets sent out or else it won’t get taken* (HSC2A).

Two HSCs temporarily suspended food distribution services for one week due to a staff member testing positive for COVID-19.

#### 3.2.2. Category 2: Changes in Food Distribution Process

EFAOs made two major changes to the food distribution process, including change from client-choice to pre-packaged boxes, and registration process changes.

##### Change from Client-Choice to Pre-Packaged Boxes

With the adoption of a drive-through model, eleven EFAOs (seven HSCs and four FPs) reported changing from a client-choice model (where the clients choose their foods) to a pre-boxed model to reduce physical contact and increase efficiency: *What we normally do out here, we have the client choice option and so everybody normally comes in and they do their shopping, and they pick and choose…That went well for about 20 min and then it started backing up! So, I decided, you know what, we’re just going to go to a pre-packed box* (HSC4A).

Despite increases in demand, four HSCs offered their clients a small amount of choice while still mainly distributing pre-packaged boxes. One directly asked questions such as: *what kind of bread do you like? What kind of desserts do you want? Some extra stuff like pickles or whatever…so there is some kind of choice* (HSC5A). The other three used a preference-listing approach where the staff would try to include client choices plus the standard pre-made food package. 

Client dietary restrictions were a particular challenge for one HSC: We used to have them fill out a form about any special foods we had to keep in mind but…that took too long. We switched to having them write down and display in their closed car window for us what their dietary restrictions were. (HSC3A).

Of the six EFAOs (two HSCs and four FPs) still able to provide a full client-choice model of food distribution, only one was able to continue without modification to its pre-pandemic food distribution model, because they relied mostly on mobile FPs held outdoors with minimal staff contact.

Two HSCs abandoned the drive-through model because of space or man-power issues: we just don’t have a large parking lot and our space is limited. So we would have a lot of traffic jams and it wasn’t very organized…we decided to go back to the store but with safety precautions in place (HSC6A). 

##### Changes in Registration Process

All clients receiving assistance from an EFAO are required to register. Prior to the pandemic, several EFAOs required a paper application or face-to-face appointment. Notably, most participating HSCs conducted client registration at their social services office not at their FP. During the pandemic, EFAOs reduced client contact, and for five HSCs, the food distribution team handled registration. One HSC registered clients through text messaging: *And then the Houston Food Bank gave us—it’s called Mobilize Us—where you can send a text message and we just hand them the form and they follow the instructions, and they register themselves* (HSC7A).

Two utilized FP volunteers for the registration process: *Pre-COVID, the client would have to schedule an appointment in social services* ……, but once COVID started, we couldn’t do that since we were seeing everybody remotely. *So then we came up with the application where the food pantry just had to fill it out* (HSC1A). Two HSCs opted to keep requiring separate registration appointments with social services except in cases such as food emergencies or homeless clients.

#### 3.2.3. Category 3: Changes in Volunteerism and Staffing

Most EFAOs experienced changes in the number of volunteers; some requested federal personnel assistance. 

##### National Guard and AmeriCorps

During the first few months of the pandemic, due to significant increase in clientele, two HSCs requested government assistance. Ten AmeriCorps members helped one HSC facilitate food distribution: *During COVID…April through August, I was able to obtain 10 AmeriCorps members when all our volunteers stopped coming in* (HSC2A). The HFB re-distributed several members of the national guard to EFAO partners in need. 

##### Changes in Volunteers and Staffing

Most EFAOs (*n* = 16) had less than three paid staff members in charge of food distribution; all EFAOs and HFB reported being almost completely dependent on volunteers for food distribution. 

Seven HSCs had difficulty getting volunteers (Table 3); three reported losing corporate volunteers. One stated: *most volunteers were mainly corporate workers. They are no longer allowed to come by because of their companies* (HSC2A). Another cited a shortage in younger volunteers once schools reopened in their area.

Five EFAOs were considerably affected by the loss of volunteers aged 65+: You know, most of my volunteers here at the food pantry are advanced age or have since retired so when this first started, several of my volunteers decided to stay home…because of their age and some of their health conditions. So, I want to say the first month we were really lacking volunteers (HSC4A). All of these five EFAOs cited COVID safety concerns: Most of volunteers were seniors so most left because of their high COVID risk (FP13M). Volunteers are seniors so they were afraid to return (HSC14M).

Although three FP and two HSCs reported an initial drop in volunteers, eventually they were able to replace them, resulting in no net change in number of volunteers. All three FPs and one HSC partially achieved this by shifting volunteers from other departments. 

Three HSCs furloughed staff at the beginning of the pandemic due to closing of their thrift stores, which were alternate revenue sources. For example: *Just because there wasn’t any business at the end of February, [redacted] was released and went on unemployment* (HSC5A). As food demand stabilized at higher numbers closer to the end of 2020, four EFAOs (three FPs and one HSC) reported having to hire more staff to keep up with rising demand. 

The majority (*n* = 11) of EFAOs did not experience any changes in staff. 

#### 3.2.4. Category 4: Changes in the Amounts of Food Distributed

The amount of food distributed per person was reported to vary even before the pandemic, due to the variability in food procurement from HFB and donations. Three HSCs reported changes in the amounts of food distributed during the pandemic. One stated: *Up to two people would get a certain amount, then up to four people. Well, we had to change that, and now it’s like 1–3 and then 4–6, because just changing it by one person really changed the amount of inventory that we had, otherwise we were running out of food at the beginning* (HSC1A). 

## 4. Discussion

To our knowledge, this is the first study that identifies the internal changes in EFAOs due to COVID-19. Several factors identified in this paper should be addressed in the future to better manage the increase in EFAO utilization during times of crisis.

The CDC provided EFAOs with a set of safety and operational considerations to curb the spread of COVID-19. These include increased cleaning and disinfection, social distancing, mask usage, and outdoor food distribution [6]. Additionally, TEFAP issued COVID-19 waivers to signature requirements when registering or picking up food, allowing EFAOs to practice social distancing during the client registration process [10]. All EFAOs participating in this study reported full compliance to these recommendations, even when forced to move services indoors. Adhering to CDC [11] as well as TEFAP recommendations [10], most EFAOs utilized pre-packaged boxes and/or drive-through model to maintain social distancing. It is notable that some participants chose to implement masking and cleaning procedures in early March, well before the CDC officially recommended mask usage in April 2020 [11]. This demonstrates EFAOs being proactive by taking measures towards safety of their clients, volunteers, and employees. HSCs cited three safety precautions not undertaken by any FPs, possibly due to the differences in the size and available resources of the EFAOs. These precautions included quarantining food items, switching to appointment-only food distribution models, and increased airflow when outdoor food distribution was not possible. Findings highlight the need for individualized action plans that considers each EFAO’s space constraints, safety measures, and increased workloads on staff and volunteers.

Volunteers, who comprise most of the workforce, are pivotal to the operations of all EFAOs. There did not seem to be a predictable pattern in volunteer fluctuations, suggesting internal culture and individual needs of EFAOs were determining factors in volunteer loss and retention. Staffing is purported to have remained mostly intact due to EFAOs having a minimum number of employees to maintain operations. Thus, workforce shedding occurred only due to revenue loss or loss of volunteers. Gains in additional staff, volunteers, or federal personnel mirrored large increases in food demand. A loss of volunteers was also reported by the HFB citing difficulties finding safe ways to social distance inside the distribution warehouse (a difficulty echoed by EFAOs) [5].

Though grants were reported to have been made available, the combination of volunteer losses, increased demand, and food availability fluctuations caused financial strain for some EFAOs. Despite these challenges, most EFAOs chose to retain some degree of choice for clients. Though not specifically measured in this study, loss of client-choice distribution models is very likely to have disproportionately affected food insecure households with special dietary needs; further research is warranted to assess the full impact.

EFAOs are not only important entities in the food safety net, but also one of the first local resources utilized during economic insecurity. Challenges highlighted in this paper should be addressed in order for EFAOs to continue to operate safely and effectively. First, a more stable staffing pool is needed during times of crises to meet rising demand and cope with natural losses of volunteers. An opportunity exists for a simple, electronic client registration process to allow EFAOs to reallocate volunteers and staff to more vital food distribution tasks and further reduce the need for contact as long as social distancing remains a necessity. Lastly, several participants mentioned funding affecting staffing decisions. 

A strength of this study was the use of qualitative methods to explore an understudied issue that is relevant today as well as in the future. Another strength was the use of two trained coders acting independently. The limitations of this study highlight opportunities for future research. A key limitation of this study was a small sample size. However, we did obtain theoretical saturation, which makes this less of a concern. Another limitation was that the study was conducted in a limited geographic region of the US using convenience sampling, reducing the generalizability of the findings. Interviews also did not focus on the myriad of other social services that HSCs provide to the community, which indirectly affect the food distribution branch of the sites involved. The timeframe in which the interviews were conducted preceded wide vaccine availability to the public. Vaccine attitudes and compliance among EFAOs were therefore not addressed and would be a point of interest in follow-up studies.

Despite these limitations, this study adds to the growing body of research on how COVID-19 impacted the food safety net in the US. It illustrates the diverse ways in which COVID-19 affected both large HSCs and smaller FPs. It highlights the vulnerability of many of these organizations and provides a useful starting point for further research.

## 5. Conclusions

EFAOs play an important role in the food safety net. Despite their importance, the changes acquired by EFAOs due to the COVID-19 pandemic have been largely unexplored in current literature. Our findings suggest special attention needs to be directed towards individualizing safety recommendations, meeting increased food demand, and alleviating staffing needs in EFAOs to meet increased usage during COVID-19 and other future crises. Future research is needed to quantify the full extent of change experienced in staffing and volunteers, food items, and funding by EFAOs as a result of COVID-19, as well as to explore any interventions aimed at managing the challenges highlighted in this paper.

## Figures and Tables

**Table 1:** List of categories and subcategories.

Categories	Subcategories
Category 1: Safety measures adopted	Social distancing
	PPE (including masks)
	Health screenings
	Increased cleaning
	Other safety measures
Category 2: Changes in food distribution process	Client-choice to pre-packaged box
	Changes in registration process
Category 3: Changes in volunteerism and staffing	National Guard and AmeriCorps
	Changes in number of volunteers
	Changes in staffing
Category 4: Changes in the amounts of food distributed	None

**Table 2:** Frequency of reported safety measures taken during food distribution by types of EFAOs.

	Human Service Centers (*n* = 10)	Stand-Alone Food Pantries (*n* = 8)	Overall (*n* = 18)
	*n* (%)	*n* (%)	*n* (%)
Social Distancing	10 (100)	8 (100)	18 (100)
PPE (including masks)	10 (100)	8 (100)	18 (100)
Health screenings	5 (50)	6 (75)	11 (61)
Increased cleaning	7 (70)	3 (38)	10 (56)
Other safety measures	4 (40)	1 (13)	5 (28)

**Table 3:** Overall changes in numbers of staff and volunteers during COVID-19 by type of EFAOs.

	Human Service Centers (*n* = 10)	Stand-Alone Food Pantries (*n* = 8)	Overall (*n* = 18)
	*n* (%)	*n* (%)	*n* (%)
**Volunteers**			
Increase	3 (30)	3 (38)	6 (33)
Decrease	5 (50)	2 (25)	7 (39)
No change	2 (20)	3 (38)	5 (28)
**Staff**			
Increase	2 (20)	2 (25)	4 (22)
Decrease	2 (20)	1 (13)	3 (17)
No change	6 (60)	5 (63)	11 (61)

## Data Availability

The datasets generated and/or analyzed during the current study are not publicly available due to concerns regarding privacy but select data are available from the corresponding author on reasonable request.

## Supplementary Materials

The following are available online at https://www.mdpi.com/article/10.3390/nu13124267/s1, Table S1: Interview questions.

## References

[1] Gundersen C., Hake M., Dewey A., Engelhard E. (2021). Food insecurity during COVID-19. Appl. Econ. Perspect. Policy.

[2] Feeding America The Impact of the Coronavirus on Food Insecurity in 2020 & 2021. https://www.feedingamerica.org/sites/default/files/2021-03/National%20Projections%20Brief_3.9.2021_0.pdf.

[3] Houston Food Bank About US—Houston Food Bank. https://www.houstonfoodbank.org/about-us/.

[4] Food and Nutrition Service—U.S Department of Agriculture. TEFAP Fact Sheet. https://www.fns.usda.gov/tefap/tefap-fact-sheet.

[5] Dellinger H. Hungry Houstonians Turn to Food Bank for Help during COVID-19 Pandemic. https://www.houstonchronicle.com/news/houston-texas/houston/article/covid-pandemic-hunger-food-bank-houston-texas-15787932.php.

[6] Centers for Disease Control and Prevention COVID-19: Considerations for Food Pantries and Food Distribution Sites. https://www.cdc.gov/coronavirus/2019-ncov/community/organizations/food-pantries.html.

[7] Archibald M.M., Ambagtsheer R.C., Casey M.G., Lawless M. (2019). Using Zoom videoconferencing for qualitative data collection: Perceptions and experiences of researchers and participants. Int. J. Qual. Methods.

[8] Braun V., Clarke V., Hayfield N., Terry G., Liamputtong P. (2018). Thematic Analysis. Handbook of Research Methods in Health Social Sciences.

[9] Fusch P.I., Ness L.R. (2015). Are we there yet? Data saturation in qualitative research. Qual. Rep..

[10] Food and Nutrition Service—U.S Department of Agriculture. TEFAP COVID-19 Q&As. https://www.fns.usda.gov/tefap/covid-19-qas.

[11] Dwyer C., Aubrey A. CDC Now Recommends Americans Consider Wearing Cloth Face Coverings in Public. https://www.npr.org/sections/coronavirus-live-updates/2020/04/03/826219824/president-trump-says-cdc-now-recommends-americans-wear-cloth-masks-in-public.

