# Supplementary material for: The Impact of the COVID-19 Pandemic on Food Distribution at Emergency Food Assistance Organizations in the Southwestern United States: A Qualitative Investigation
Source: Nutrients. 2021 Nov 26;13(12):4267. doi: 10.3390/nu13124267 (PMC8703558; doi:10.3390/nu13124267)
Supplement: Supplementary file 1 [file nutrients-13-04267-s001.zip › nutrients-1428804-supplementary.pdf]

The impact of COVID-19 pandemic on food distribution at emergency food assistance organizations in the Southwestern United States: A qualitative investigation

Table S1. Interview questions.

1. The COVID-19 pandemic has changed our lives and have impacted our daily activities. Please tell us if your organization ever shut down shut down? If yes…
   A. For how long?
   B. When did it re-open?
   C. What were some of the challenges, if any, that you experienced during re-opening of the pantry?

2. How would you describe the current operation of your organization?
   A. What, if any, safety guidelines/procedures are you following for employees, volunteers and clients?
      i. For overall operations
      ii. For food distribution
      iii. For food procurement
      iv. For other services
   B. How, if at all, are these guidelines/procedures different from beforeCOVID-19?
   C. How many days in a week is the pantry open?
      i. For overall operations
         a. Change in the number of days due to COVID-19
         b. Change in the number of hours per day
      ii. For food distribution
         a. Change in the number of days due to COVID-19
         b. Change in the number of hours per day
   D. How many staff are present at the pantry?
      i. For overall operations
         a. How has this changed, if at all?
            1) Number of staff
         b. Type of staff
            1) Employees
            2) Volunteers
            3) Others
      ii. For food distribution
         a. How has this changed, if at all?
            1) Number of staff
         b. Type of staff

1) Employees
2) Volunteers
3) Others

E. Considering all that has happened over the past 7 months, what factors, if any, affect your current overall operations?

i. Funding
ii. Food donations
iii. Availability of staff (employees/volunteers)
iv. Food waste
v. Expenses towards preventive measures for COVID-19 (masks, gloves, cleaning supplies, etc.)
vi. Other

---

3. How would you describe the current food distribution process?

A. How, if at all, is the current food distribution process different than prior to COVID-19?

B. What are the most common foods currently distributed?

i. What are the contents of a typical food package?
   a. Type/variety
   b. Amount

ii. What, if any, are the differences in the foods distributed currently and prior to COVID-19?
   a. Type/variety
   b. Perishable/non-perishable
   c. Amount
   d. Frequency of clients receiving foods
   e. Why have these changes been necessary?

C. Considering all that has happened over the past 7 months, what factors, if any, affect your current food distribution process?

i. Funding
ii. Availability of staff (employees/volunteers)
iii. Food waste
iv. Food distribution time
v. Other
